# Role of freshwater floodplain-tidal slough complex in the persistence of the endangered delta smelt

**DOI:** 10.1371/journal.pone.0208084

**Published:** 2019-01-02

**Authors:** Brian Mahardja, James A. Hobbs, Naoaki Ikemiyagi, Alyssa Benjamin, Amanda J. Finger

**Affiliations:** 1 California Department of Water Resources, Division of Environmental Services, West Sacramento, California, United States of America; 2 University of California–Davis, Department of Wildlife, Fish and Conservation Biology, Davis, California, United States of America; 3 University of California–Davis, Department of Animal Science, Genomic Variation Laboratory, Davis, California, United States of America; Tanzania Fisheries Research Institute, UNITED REPUBLIC OF TANZANIA

## Abstract

Seasonal floodplain wetland is one of the most variable and diverse habitats found in coastal ecosystems, yet it is also one of the most highly altered by humans. The Yolo Bypass, the primary floodplain of the Sacramento River in California’s Central Valley, USA, has been shown to provide various benefits to native fishes when inundated. However, the Yolo Bypass exists as a tidal dead-end slough during dry periods and its value to native fishes has been less studied in this state. During the recent drought (2012–2016), we found higher abundance of the endangered Delta Smelt (*Hypomesus transpacificus*), than the previous 14 years of fish monitoring within the Yolo Bypass. Meanwhile, Delta Smelt abundance elsewhere in the estuary was at record lows during this time. To determine the value of the Yolo Bypass as a nursery habitat for Delta Smelt, we compared growth, hatch dates, and diets of juvenile Delta Smelt collected within the Yolo Bypass with fish collected among other putative nursery habitats in the San Francisco Estuary between 2010 and 2016. Our results indicated that when compared to other areas of the estuary, fish in the Yolo Bypass spawned earlier, and offspring experienced both higher quality feeding conditions and growth rates. The occurrence of healthy juvenile Delta Smelt in the Yolo Bypass suggested that the region may have acted as a refuge for the species during the drought years of 2012–2016. However, our results also demonstrated that no single region provided the best rearing habitat for juvenile Delta Smelt. It will likely require a mosaic of habitats that incorporates floodplain-tidal sloughs in order to promote the resilience of this declining estuarine fish species.

## Introduction

Floodplain wetlands are highly dynamic environments, located at the interface of lowland-riverine and coastal ecosystems that play a crucial role in estuarine productivity [1,2,3]. During dry seasons, floodplain wetlands exist as shallow channel habitats that can act as hotspots of local primary and secondary productivity [4], sequester large amounts of carbon [5], and provide drought refugia for wildlife [6]. Meanwhile, floodplain inundation during wet seasons can increase in-situ productivity as well as deliver sediment, nutrients and productivity downstream, providing a mosaic of inter-connected riverine, floodplain and estuarine landscapes [2,7,8,9]. In coastal systems, floodplain wetlands can function as productive nursery habitats for fish exhibiting a diversity of life histories [10,11,12]. This nursery function stems from the shallow ephemeral nature of inundated floodplain wetlands, providing refuge from large predators and high densities of prey for rapid growth [8,9,13,14,15,16,17].

Despite the diverse array of benefits they provide, many floodplain wetland habitats around the world have been heavily degraded by channelization, water diversion and conversion to agricultural lands [18,19]. Moreover, the pressure to further alter wetland habitat for resource development will likely intensify as human populations continue to expand [20,21]. Given that habitat loss is one of the primary drivers of biodiversity loss and erosion of ecological resilience, additional reduction in wetland habitat is expected to have adverse effects on the biodiversity and ecological functioning of estuarine ecosystems [21]. This erosion of ecological function is further exacerbated by drought conditions, which are intensifying in the west with changing climate [22]. Due to the myriad of anthropogenic perturbations to floodplain wetland habitats, it is imperative to gain a better understanding of their ecological function and role in providing resilience to species that depend on these habitat mosaics.

Similar to other estuaries around the world, the historic floodplains and wetlands of San Francisco Estuary, California, USA (hereinafter SFE) have been largely reclaimed for agriculture and urban development [23,24]. Within the upper SFE, over 90% of freshwater wetland habitats have been lost since the 1800’s and replaced with leveed channels and deep open waters, that have little to no connectivity to former floodplain habitats [25,26]. This extensive landscape transformation, along with other changes such as the introduction of invasive species, increase in contaminant inputs, and alteration of freshwater flows to the SFE have contributed to the steep decline of multiple native fish species over the past two decades [27,28,29].

The Delta Smelt (*Hypomesus transpacificus*), a small euryhaline osmerid fish endemic to the tidal freshwater and brackish portions of the SFE, is among the fish species that has experienced a long-term decline in abundance [29,30]. Although the species was historically common in the SFE, a rapid decline in the Delta Smelt population during the 1980s resulted in its listing as threatened under the California and Federal Endangered Species Acts in 1993. Further decline in the abundance of the Delta Smelt around 2000 led to its up-listing to endangered status under the California Endangered Species Act in 2009. Today, efforts to restore tidal wetland habitats in the SFE are ongoing to help recover populations of several threatened and endangered fish species, including the Delta Smelt [31]. The Yolo Bypass, one of the largest remnant floodplain habitats in the SFE, has been specifically identified as a key area for tidal wetlands and floodplain restoration [31]. It has been hypothesized that Delta Smelt would benefit from tidal wetland restoration and environmental flows through export of lower trophic food web productivity to the surrounding environment [32]. However, previous studies on Delta Smelt have solely focused on open water habitat, where Delta Smelt have primarily been found in the past few decades [33,34,35,36]. To date, no studies have examined diet and growth of Delta Smelt in tidal wetland habitats.

In this study, we examined the growth, via otolith microstructure, and diet of Delta Smelt collected at a floodplain-tidal slough wetland complex in the Yolo Bypass to improve our understanding of how the species utilizes tidal wetland environment. More specifically, our research questions are: (i) Do juvenile Delta Smelt grow faster in a floodplain-tidal slough complex? (ii) Do juvenile Delta Smelt in floodplain-tidal slough complex hatch earlier in the year than other areas? (iii) What are the primary prey items for Delta Smelt in a floodplain-tidal slough complex such as the Yolo Bypass? We hope to provide insight into the potential impact of future tidal wetland habitat restoration in the SFE by answering these study questions.

## Methods

### Ethics statement

Fish collection for this study was done under the United States Endangered Species Act Section 7 Biological Opinion (file number 1-1-96-F-1 and 1-1-98-I-1296) and the California Scientific Collection Permit #10842.

### Study area

**Fig 1:**
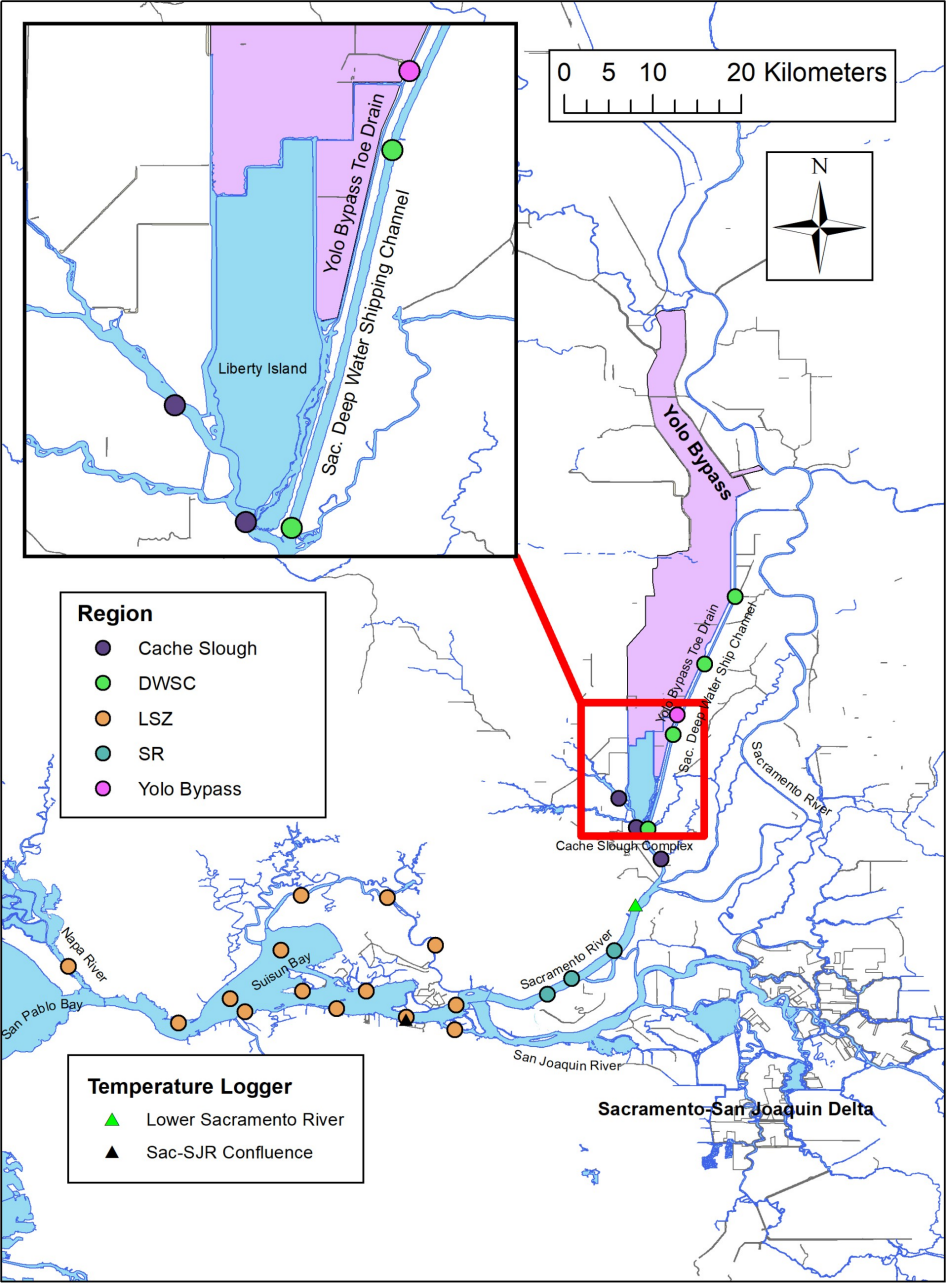
Map of the upper San Francisco Estuary depicting the sites at which Delta Smelt was collected, their region classifications, and the locations of temperature loggers. DWSC = Sacramento Deep Water Ship Channel, LSZ = low salinity zone, SR = Sacramento River. Blue colored region indicates water bodies (perennially wetted area) in the estuary and purple colored region denotes the extent of Yolo Bypass floodplain that can be inundated during flooding events.

The SFE is one of the largest estuaries on the west coast of the United States. The estuary includes San Francisco Bay to the west, which is heavily influenced by marine waters, the brackish Suisun Bay region in the middle, and the tidal freshwater complex of the Sacramento-San Joaquin Delta to the east (Fig 1). Comprising a large portion of the northern region of the Sacramento-San Joaquin Delta is the Yolo Bypass. The Yolo Bypass is the primary floodplain of the San Francisco Estuary (approximately 61 km long and 240 km^2^), and has been engineered as a partially leveed basin to convey the majority of Sacramento River flow during high water events in winter and spring [37]. During the summer and early fall, the wetted area in the Yolo Bypass is mainly restricted to the “Toe Drain” a narrow (≤ 50 m wide) and shallow (≤ 5 m deep) tidal dead-end slough along the eastern edge of the bypass that empties the floodplain and provides agricultural water supply. During these drier periods, the flow within the Yolo Bypass Toe Drain is largely influenced by tides and agricultural discharges upstream. As a result, the Yolo Bypass Toe Drain has extended periods of net negative outflow at times in the summer and fall due to local water diversions. Connectivity between the Yolo Bypass and the lower San Francisco Estuary is maintained through the Cache Slough complex, a turbid backwater area adjacent to the Yolo Bypass that also contains various dead-end tidal sloughs and fringe marsh habitat [38].

### Field sampling

The California Department of Water Resources’ Yolo Bypass Fish Monitoring Program (YBFMP) has conducted fish monitoring in the Yolo Bypass since 1998 [39,40, 41]. For up to seven days a week between January and June, the YBFMP operates a 2.5-meter rotary screw trap located in the Yolo Bypass Toe Drain to sample out-migrating juvenile fishes. The rotary screw trap is checked daily and any fish over 25 millimeters (mm) in fork length is identified to species and measured for length in the field. The YBFMP generally observes adult Delta Smelt between February and April and juvenile Delta Smelt between May and July [42]. Delta Smelt collected by the YBFMP for this study were collected from mid-May through June, assigned a unique fish identification number in the field, measured for fork length to the nearest 1-mm and preserved in 95% ethanol.

Juvenile Delta Smelt were also collected throughout the San Francisco Estuary by the California Department of Fish and Wildlife’s Summer Townet Survey [43]. The Summer Townet Survey samples juvenile and smaller-sized fishes using a 2.5 mm mesh conical net from June to August each year at multiple locations from San Pablo Bay to the upstream portion of the Sacramento-San Joaquin Delta. In this study, we focused on Delta Smelt collected by the Summer Townet Survey in the month of June between 2010 and 2014 for comparison with fish collected by the YBFMP. Delta Smelt collected by the Summer Townet Survey were assigned a unique fish identification number in the field, measured for fork-length to the nearest 1-mm and either preserved in 95% ethanol (2010–2011) or frozen in liquid nitrogen (2012–2014) (Table 1). Although several Delta Smelt were captured at locations outside of Yolo Bypass in June of 2016 (S1 Fig), these fish were either released back into the water or preserved in formalin that typically bias further otolith analysis [44]. For Delta Smelt size and catch number comparison, we also used data collected from the California Department of Fish and Wildlife’s 20-mm Survey, a monitoring program that samples post-larval and early juvenile fishes using 1.6 mm mesh conical plankton net from March to August each year at various locations around the San Francisco Estuary [45]. For fish growth and hatch date comparisons between Yolo Bypass and the rest of the San Francisco Estuary, we focused on juvenile Delta Smelt (defined as fork length <60 mm) because we expect juvenile fish conditions to generally reflect the regions in which they were captured due to their reduced mobility relative to adults.

**Table 1:** Number of juvenile Delta Smelt analyzed for otolith microstructure in this study, sorted by region and year. All fish collected at the Yolo Bypass were genetically confirmed as pure Delta Smelt. Cache is Cache Slough region, DWSC is Sacramento Deep Water Ship Channel, LSZ is low salinity zone, SR is Sacramento River, and Yolo is Yolo Bypass. Numbers in parentheses indicate the number of fish that were also processed for diet analysis, followed by the number of fish with stomach content intact.

Year	Region				
	Cache	DWSC	LSZ	SR	Yolo, YBFMP
2010	0	0	21	3	2 (2,2)
2011	13	19	38	1	0
2012	13	36	25	8	6 (6,4)
2013	9	24	35	9	15 (15,11)
2014	0	22	0	27	5 (5,5)
2015	1	3	0	0	34 (34,20)
2016	0	0	0	0	6

### Study species

Delta Smelt is an annual fish species that typically exhibit a semi-anadromous life history. Young-of-year Delta Smelt are spawned in freshwater during springtime, but migrate downstream to the low salinity portion of the estuary (~1 to 6 parts per thousand) fairly quickly during late spring and early summer [29,43]. The majority of the species remain in this low salinity zone throughout most of the year and migrate upstream to freshwater for spawning when flows increase during the winter [46]. The low salinity zone of the San Francisco Estuary is typically located between the Suisun Bay and the confluence of the Sacramento and San Joaquin Rivers (Fig 1) depending on the amount of freshwater flow into the Sacramento-San Joaquin Delta. In the past several years, the numbers of juvenile and adult Delta Smelt observed in the Yolo Bypass Toe Drain, a perennially freshwater habitat, have increased [42]. Sommer et al. [47] noted that a portion of the Delta Smelt population may remain in freshwater throughout their entire lives, but it was unclear if a freshwater floodplain-tidal wetland complex such as the Yolo Bypass represents a suitable or fringe habitat for Delta Smelt.

### Genetic assignment

The Wakasagi (*Hypomesus nipponensis*), an introduced osmerid species in the San Francisco Estuary, can sometimes co-occur and hybridize with the Delta Smelt. Hybrids of the two osmerid species were previously found in the Yolo Bypass [48]; however, the two species are morphologically similar and accurate identification of hybrids by morphology alone may not be feasible [49]. Moreover, a large portion of juvenile osmerids that the YBFMP collected were partially degraded, leading to possible misidentifications. Therefore, the YBFMP has conducted genetic species identification on all osmerid fish collected and preserved by the program since 2010 [50]. For all genetic samples, 24 single nucleotide polymorphism (SNP) assays were used to determine the species or hybrid status of each fish. Only fish genetically assigned as pure Delta Smelt were used in this study. Details on the assay development and genetic assignment method can be found in Benjamin et al. [50].

### Otolith microstructure

Sagittal otoliths were dissected from the fish cranium using ultra-fine forceps (Dupont SE140, stainless steel) and stored dry in tissue culture trays. Before mounting, otoliths were “cleared” by soaking in 95% ethanol for up to 24 hours. Otoliths were then mounted onto glass slides with Crystal Bond thermoplastic resin in the sagittal plane, ground to the core on both sides with 1,200 grit wet-dry sandpaper and polished with a polishing cloth and 0.3-micron polishing alumina on polishing wheel (MIT Corp). Otoliths were digitized with a 12-Megapixel digital camera (AM Scope) at a magnification of 20X with an Olympus CH30 compound microscope. Digital images at 20X magnification were merged into a complete image of a transect from the core to the dorsal edge (Adobe Photoshop) at a 90° angle from the primary axis of the otolith.

In a previous study, otolith increments in Delta Smelt were determined to form daily and accurately represent age [51]. Otolith daily increments were enumerated to estimate age using calibrated images in Image-J 4.0 (United States National Institutes of Health; https://imagej.nih.gov/ij/). Aging was conducted by two readers and evaluated for age agreement using the Average Percent Error (APE). If the APE between readers for an individual was greater than 10%, a third reading was done by a senior age reader and the age reading most dissimilar was discarded.

Individual growth rate (G) for each fish was calculated by:

$$G=\frac{{Length}_{at\;capture}-{Length}_{at\;hatch}}{Mean\;Age}$$

Where age was the mean number of otolith increments from multiple age readings and length at hatch was assumed to be 5.2 mm, the mean size at hatch from the captive Delta Smelt population maintained by the UC Davis Fish Conservation and Culture Laboratory [51]. The hatch date for each fish was calculated by subtracting the mean age from the capture date and was reported as the number of days from January 1^st^.

### Diet composition

A subset of the genetically-verified juvenile Delta Smelt collected at the Yolo Bypass were examined for diet composition (N = 63) (Table 1). The intact stomach was removed from each individual fish and stored in 10% buffered formalin. Stomach contents were then removed, sorted to the lowest taxonomic level possible (depending on the digestive state), enumerated, and weighed. Food matter that was too digested to be identified to any taxon was categorized under “unidentified”. Because fish were collected via rotary screw trap, fish were often found dead for an unknown period of time making a more detailed assessment of feeding success uncertain. A total of twenty-one samples had empty or damaged stomachs and were removed from further diet analysis.

### Data analysis

We used ordinary least squares (OLS) linear regression to confirm that there was a strong relationship between fish age and size, and that the relationship is linear for the size range evaluated in this study. We subsequently used a generalized linear mixed model (GLMM) to evaluate if G and hatch date for Delta Smelt vary by region of capture. The regions considered were Cache Slough, Sacramento Deep Water Ship Channel, low salinity zone, lower Sacramento River, and the Yolo Bypass (Fig 1). Two GLMMs were fitted, one with G as the response variable, and another with hatch date as the response variable. For both GLMMs, region was included as a dummy fixed effect with Cache Slough as a baseline, and to account for the unequal representation of each region by year, year was added as a random effect. All models were fit using either the base package (OLS) or lme4 package (GLMM) in R with identity link and Gaussian error distribution [52,53]. Pairwise differences of G and hatch date between regions were evaluated using Tukey pairwise comparison test under the multcomp R package [54] with α = 0.05 and Bonferroni-Holm correction.

We summarized the diet composition information by calculating the frequency of occurrence, percent by number, and percent by weight for each prey taxon. The prey food taxa are then ranked by their index of relative importance (IRI) [55], calculated as follows:

IRI=(N+W)(O)

Where *N* is the percent by number, *W* is the percent by weight, and *O* is the frequency of occurrence.

Because water temperature is a well-known factor affecting spawning activity and growth [56], we also examined water temperature data collected at the YBFMP rotary screw trap location, a location in the lower Sacramento River region near Rio Vista (38° 9' 36.576'' N, 121° 41' 7.08'' W), and a location near the confluence between Sacramento and San Joaquin Rivers (38° 2' 35.16'' N, 121° 55' 8.292'' W) during the Delta Smelt spawning period (February to May) for the study period (2010–2016). Continuous water temperature data at the YBFMP rotary screw trap location was collected using HOBO Pro v2 Water Temperature Data Logger (Onset Corp), while temperature data from the lower Sacramento River and Sacramento-San Joaquin Rivers confluence was collected using YSI-6600 V2 multi-parameter Water Quality Sonde. Using daily average water temperature data, we calculated for each year and station the Delta Smelt spawning window length (number of days when average temperature is between 15 and 20°C) [56,57], median day of year for the spawning window, and the February-May mean temperature.

## Results

### Catch pattern

**Fig 2:**
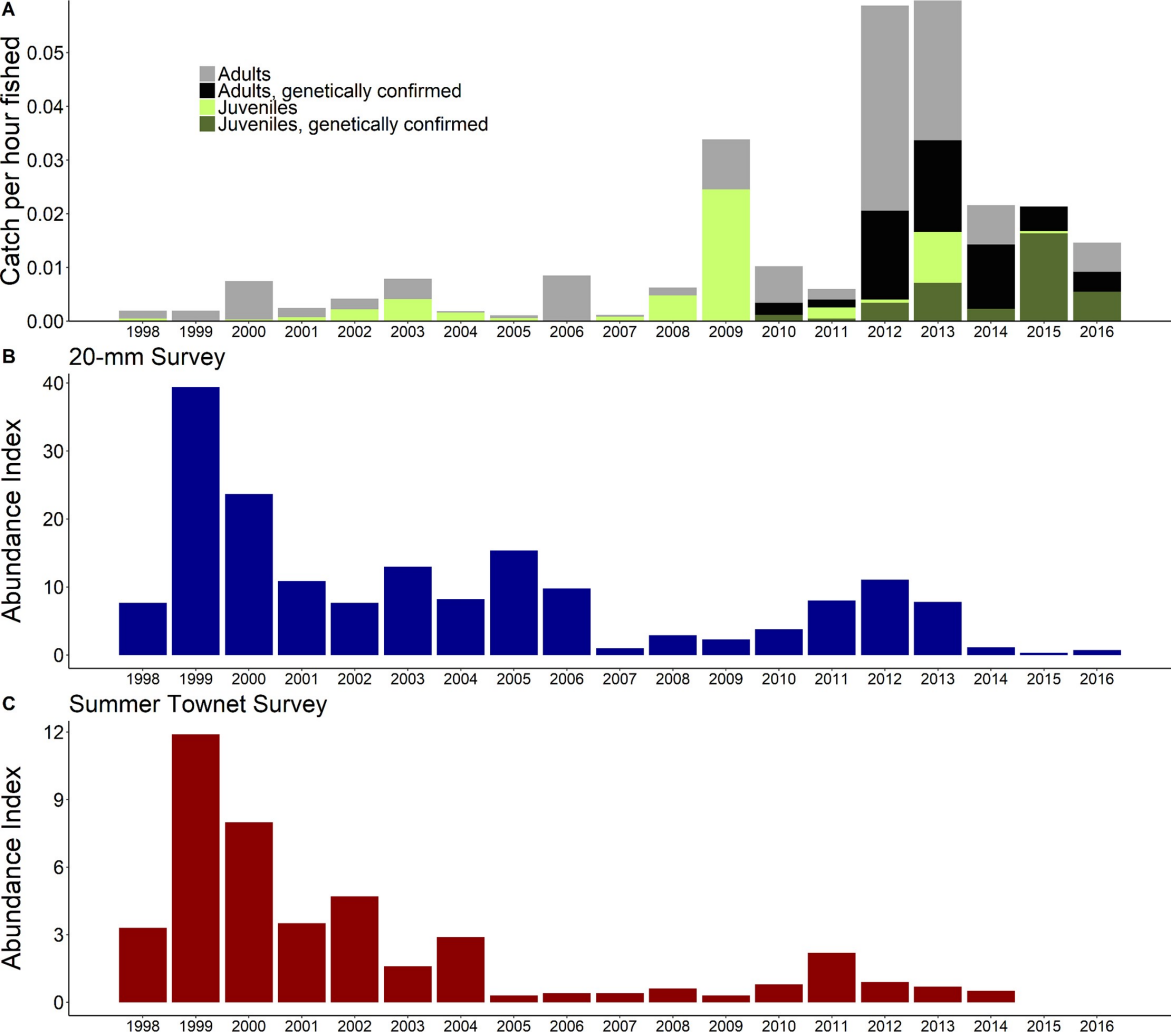
Catch per unit effort of Delta Smelt in the Yolo Bypass compared to elsewhere in the San Francisco Estuary: a.) Catch per hour of Delta Smelt in the Yolo Bypass rotary screw trap sorted by calendar year, life stage, and whether fish was genetically confirmed as Delta Smelt. b.) Abundance index from the 20-mm Survey [45]. c.) Abundance index from the Summer Townet Survey [43].

**Fig 3:**
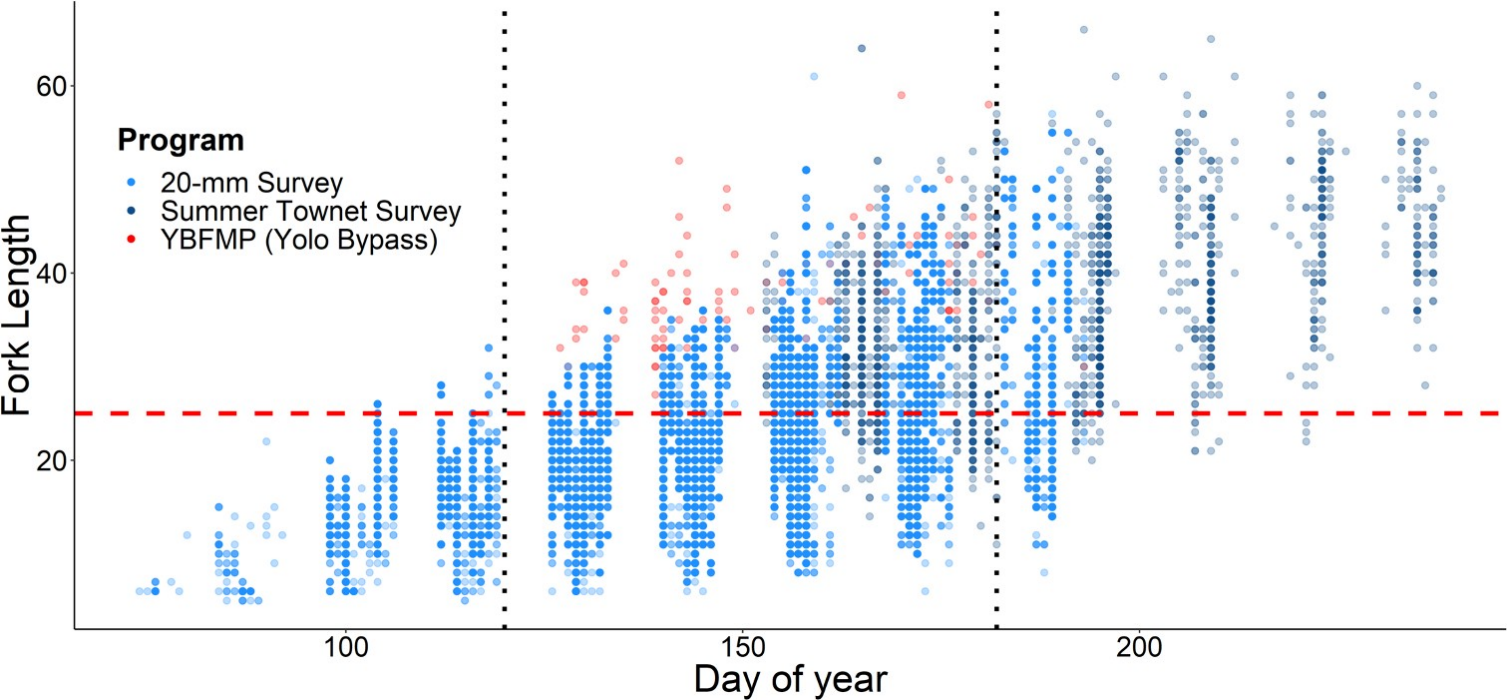
Comparison of juvenile Delta Smelt size between the Yolo Bypass (red) and elsewhere in the San Francisco Estuary (from 20-mm Larval Survey in light blue, from Summer Townet Survey in dark blue). Note that the YBFMP does not record fish with fork length under 25 mm. Red dashed line represents the 25 mm fork length lower limit cutoff for the YBFMP. Areas between the vertical black dotted lines represent the months of May and June, the months of focus for this study.

A total of 369 Delta Smelt were captured by the YBFMP rotary screw trap at the Yolo Bypass Toe Drain between 2010 and 2016, of which 68 were juveniles and genetically confirmed to be pure (non-hybridized) Delta Smelt. In contrast to the rest of the San Francisco Estuary, we observed a higher number of Delta Smelt in the Yolo Bypass in recent years (2010–2016) relative to the late 1990s and early 2000s (Fig 2). Juvenile Delta Smelt in the Yolo Bypass also seemed to be larger in size during the late summer compared to elsewhere in the San Francisco Estuary (Fig 3). It is important to note however, that the YBFMP does not count or measure fish smaller than 25 mm fork length, and typically cease their sampling at the end of June. When considering only fish longer or equal to 25 mm fork length in the months of May and June for the study period (2010–2016), the juvenile Delta Smelt found in the 20-mm Survey, Summer Townet Survey, and the YBFMP had fork length (mean ±SD) of 29.5 ±4.7, 33.8 ±6.5, and 37.5 ±6.2 mm, respectively.

### Growth and hatch date

**Fig 4:**
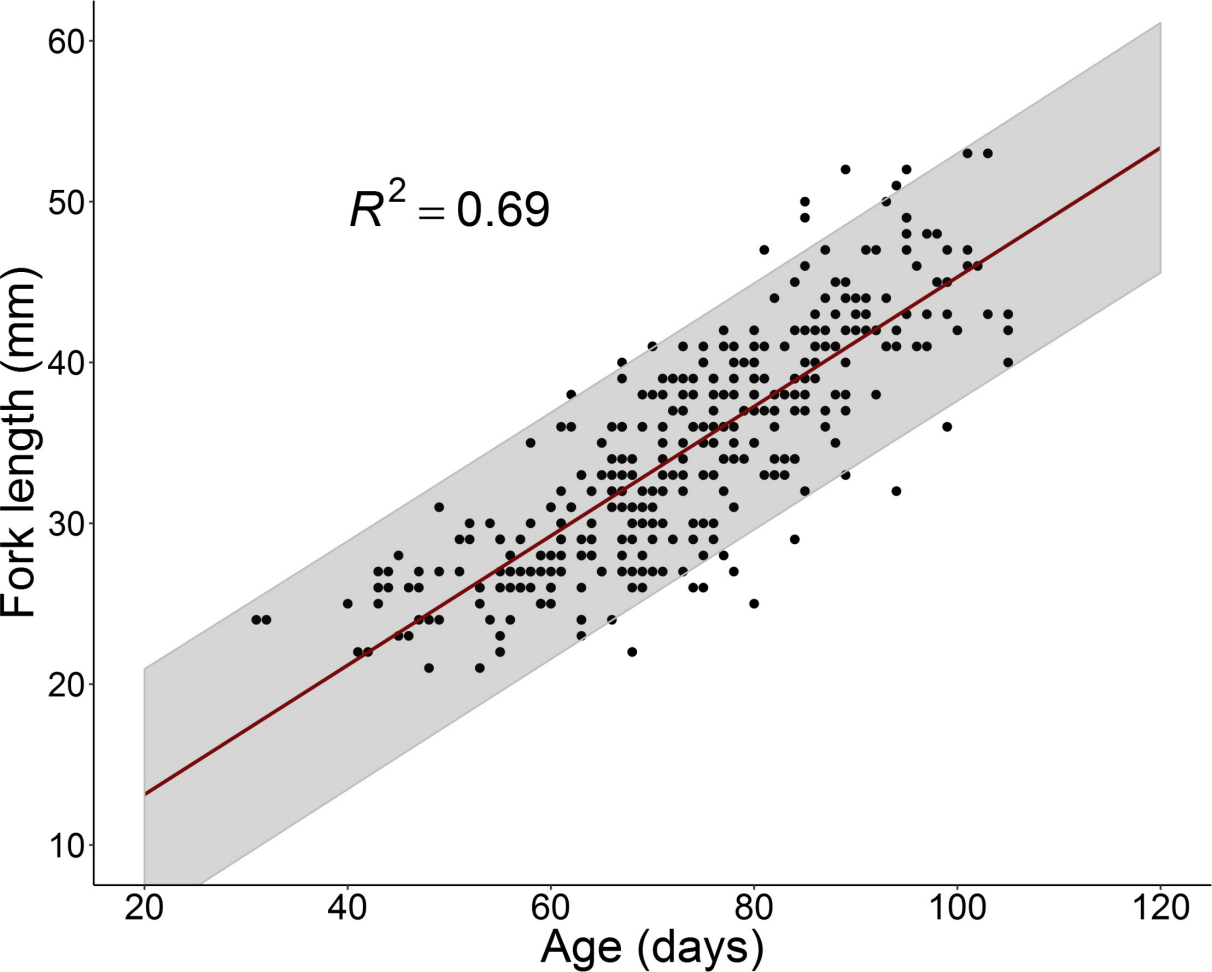
OLS regression results demonstrating the linear relationship between fish age and length in our dataset. Each dot represents a single individual fish and shaded region represent the 95% prediction interval of the regression model.

All genetically confirmed juvenile Delta Smelt from the Yolo Bypass and 307 juvenile Delta Smelt collected by the Summer Townet Survey were examined for daily age and growth rate and hatch date. Overall APE for multiple age readings of all Delta Smelt otoliths was 2.4%, corresponding to mean daily age difference of 4 days. We found a positive linear relationship between age and length (Fig 4; R^2^ = 0.68, p < 0.001). Null results from the OLS regression relating G and age provided further evidence that growth for juvenile Delta Smelt in this study is linear (R^2^ < 0.01, p = 0.26).

**Fig 5:**
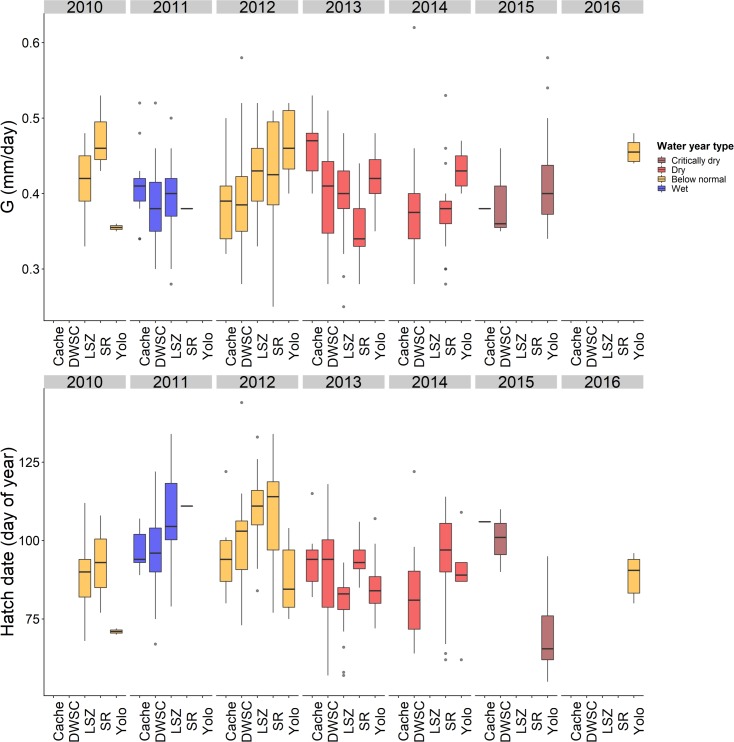
Box plots showing G (individual growth rate) and hatch date for juvenile Delta Smelt used in this study by region and year. Color indicates the water year classification for the year (e.g. critically dry, dry, wet, etc.). Region codes are as follows: Cache = Cache Slough region, DWSC = Sacramento Deep Water Ship Channel, LSZ = low salinity zone, SR = Sacramento River, Yolo = Yolo Bypass.

**Fig 6:**
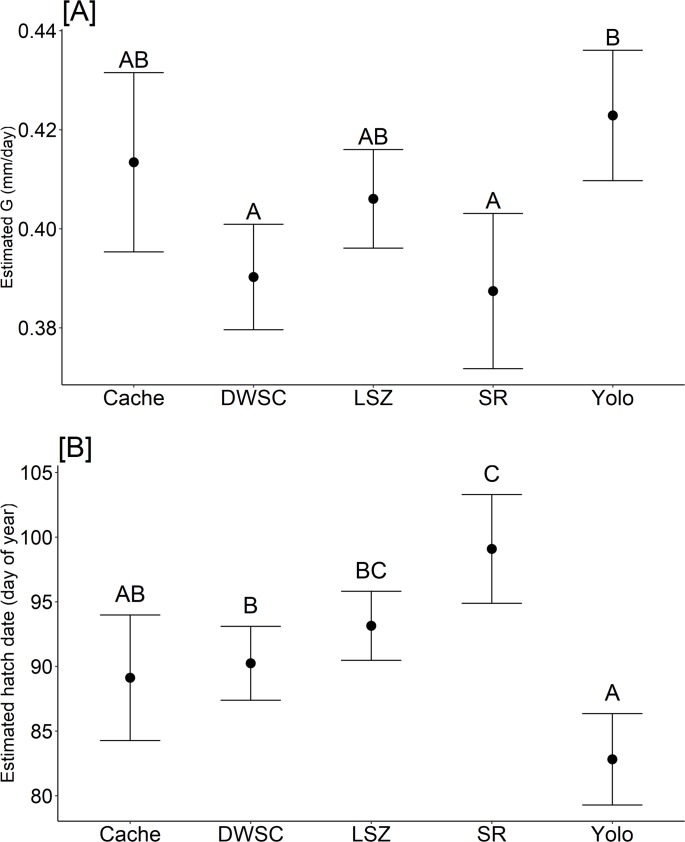
Estimated mean a.) individual growth rate and b.) hatch date by region based on our GLMM. Error bars are the 95% confidence intervals for each region. Letters above error bars denote grouping with significant differences at α = 0.05 after Bonferroni-Holm correction (e.g. A and B regional difference was statistically significant, and as such regions labeled AB were not statistically different from regions labeled as either A or B). Region codes are as follows: Cache = Cache Slough region, DWSC = Sacramento Deep Water Ship Channel, LSZ = low salinity zone, SR = Sacramento River, Yolo = Yolo Bypass.

GLMM results indicated that G varies across regions but with a considerable amount of overlap (Fig 5, Table 2). Tukey pairwise comparison test for G was significant only for two out of ten regional comparisons (p < 0.05): Yolo Bypass and Sacramento Deep Water Ship Channel, and Yolo Bypass and lower Sacramento River. Yolo Bypass showed higher G for both cases (Fig 6A). GLMM and Tukey test for hatch date found significant differences for five out of ten pairwise comparisons among regions (p < 0.01): Lower Sacramento River and Cache Slough region, lower Sacramento River and Sacramento Deep Water Ship Channel, lower Sacramento River and Yolo Bypass, Yolo Bypass and Sacramento Deep Water Ship Channel, and Yolo Bypass and the low salinity zone. Fish found in the lower Sacramento River were born later compared to those in the Cache Slough region, Sacramento Deep Water Ship Channel, and Yolo Bypass (Fig 6B).

**Table 2:** Parameter estimates for the GLMMs used to evaluate if G and hatch date for Delta Smelt vary by region of capture. Cache = Cache Slough region, DWSC = Sacramento Deep Water Ship Channel, LSZ = low salinity zone, SR = Sacramento River, Yolo = Yolo Bypass.

Model	Parameter		Estimate (standard deviation)	Standard error
G (individual growth rate)	Fixed effects	Intercept (Cache)	0.414	0.010
		DWSC	-0.023	0.011
		LSZ	-0.008	0.010
		SR	-0.026	0.013
		Yolo	0.009	0.012
	Random effect	Intercept (year)	3.57 x 10^−5^ (0.006)	
Hatch date	Fixed effects	Intercept (Cache)	89.125	3.989
		DWSC	1.120	2.581
		LSZ	4.021	2.536
		SR	9.966	3.172
		Yolo	-6.307	3.172
	Random effect	Intercept (year)	69.93 (8.363)	

### Diet composition

Out of the 63 Yolo Bypass samples processed for diet analysis, 21 had empty or broken stomachs and were removed from further diet analysis. The calanoid copepod *Pseudodiaptomus forbesi* was the main prey item consumed by Delta Smelt in the Yolo Bypass in terms of numbers (84.34% of total prey item count), weight (73.95% of total weight), and frequency of occurrence (83.33% of analyzed samples). As expected based on their high count, weight proportion, and occurrence in the diet of Delta Smelt, *Pseudodiaptomus forbesi* had the highest index of relative importance with a value of over 13,000 (Table 3). The calanoid copepod *Sinocalanus doerrii* had the second highest index of relative importance with a value of 964.2, and the cyclopoid copepod *Acanthocyclops* spp. had the third highest with a value of 29.1.

**Table 3:** Summary of diet composition information from juvenile Delta Smelt collected in the Yolo Bypass Toe Drain (N = 42). %N is percent of prey taxon by number, %W is percent of prey taxon by weight, %FO is frequency of occurrence, and IRI is index of relative importance.

Prey taxon	Count	Weight (g)	%N	%W	%FO	IRI
*Pseudodiaptomus forbesi*	1,201	0.018	84.34	73.95	83.33	13190.79
*Sinocalanus doerrii*	124	3.0 x 10^−3^	8.71	12.61	45.24	964.16
Unidentified	12	2.3 x 10^−3^	0.84	9.66	28.57	300.19
*Acanthocyclops* spp.	40	3.0 x 10^−4^	2.81	1.26	7.14	29.07
Diaptomidae	15	4.0 x 10^−4^	1.05	1.68	4.76	13.02
*Ceriodaphnia* spp.	10	0	0.70	0	11.90	8.36
*Daphnia* spp.	4	1.0 x 10^−4^	0.28	0.42	4.76	3.34

### Water temperature comparison

Throughout the study period (2010–2016), the Yolo Bypass Toe Drain experienced higher temperatures relative to the lower Sacramento River and the Sacramento-San Joaquin Rivers confluence during the spawning months of Delta Smelt, that is between February and May (Table 4). Delta Smelt spawning window comparison among the Yolo Bypass Toe Drain, the lower Sacramento River, and the Sacramento-San Joaquin Rivers confluence also suggested that the Delta Smelt in the Yolo Bypass Toe Drain would have an earlier and shorter spawning period.

**Table 4:** Summary of February-May Delta Smelt-relevant temperature metrics comparing the Yolo Bypass Toe Drain, lower Sacramento River near the city of Rio Vista, and the confluence between Sacramento and San Joaquin Rivers (Sac-SJR confluence) for the duration of the study period. Values shown are mean temperature from February to May for each year, the number of spawning days available for Delta Smelt for each period based on temperature (15–20°C), and the median day of year for each spawning period (where January 1st = 1).

Year	February-May mean temperature (°C), with standard deviation in parenthesis			Number of spawning days available			Median day of year for spawning period		
	Yolo Bypass	Lower Sacramento River	Sac-SJR confluence	Yolo Bypass	Lower Sacramento River	Sac-SJR confluence	Yolo Bypass	Lower Sacramento River	Sac-SJR confluence
2010	15.63 (2.87)	14.20 (2.10)	13.69 (2.12)	63	52	38	114	125.5	130
2011	14.28 (4.28)	12.58 (2.50)	12.98 (2.57)	23	29	38	138	133	129.5
2012	15.35 (4.05)	14.41 (3.31)	13.99 (3.12)	18	41	44	102.5	130	130.5
2013	16.69 (3.89)	15.38 (3.63)	14.90 (3.23)	50	58	59	98.5	112.5	119
2014	17.36 (3.59)	16.34 (3.25)	15.70 (2.79)	46	49	68	89.5	109	108.5
2015	17.49 (2.57)	16.71 (2.57)	15.64 (1.83)	76	78	77	95.5	108.5	113
2016	17.37 (3.63)	15.37 (3.04)	15.42 (2.51)	48	56	57	92.5	120.5	119

## Discussion

With the onset of climate change and an increasing demand for freshwater resources, the role that floodplain wetlands play in providing nursery habitat for declining species may be larger than ever. In California, extreme hydrological events such as severe droughts or flooding are increasing due to climate change and are likely to further impact threatened and endangered species [22,58]. Our study in the Yolo Bypass occurred during a period of extreme hydrologic variability. High precipitation in winter 2011 caused significant inundation of the Yolo Bypass floodplain, followed by unprecedented drought conditions from 2012 to 2016 (S2 Fig) [29]. The overall Delta Smelt population abundance increased in 2011, but collapsed to all-time lows after 2014 (Fig 2). Despite the decline of Delta Smelt abundance elsewhere in the estuary [28,29], adult and juvenile Delta Smelt were observed in the Yolo Bypass in greater numbers during the drought than had been previously documented (Fig 2). Moreover, Delta Smelt in the Yolo Bypass fed on abundant, high quality prey, exhibited rapid growth and hatched earlier than fish found in other habitats. Our results suggest the freshwater tidal wetlands in the Yolo Bypass may provide refugium for the population during drought conditions and function as a critical nursery habitat for Delta Smelt.

The relatively high abundance and growth rates of Delta Smelt in the Yolo Bypass during recent years can be attributed to a combination of at least three factors: 1) high food density, 2) high turbidity, and 3) moderate temperature. First, calanoid copepod density from 2011–2014 was consistently higher in the Yolo Bypass than in the Sacramento River [16]. Calanoid copepods, including *Pseudodiaptomus forbesi* are the preferred prey for Delta Smelt [34], and dominated the diet composition of fish in our study. Higher availability in prey abundance has been shown to improve the growth and survival of Delta Smelt [59,60,61]. Second, Secchi depth values in the Yolo Bypass were typically within the optimal range for Delta Smelt (0.1–0.3 meters) year-round [39,40]. Turbidity is a key predictor of Delta Smelt occurrence for all life stages in the wild [43,45,62,63] and an important factor for feeding success in Delta Smelt [64,65]. Declining turbidity throughout the upper SFE has also been linked to declining catch of Delta Smelt in monitoring surveys [66,67]. Third, the Yolo Bypass also experienced slightly warmer temperature between February and May relative to other regions of the estuary (Table 4), but so far, these temperatures have remained within the physiological limits of the species to support rapid growth [68]. The elevated temperature observed in the Yolo Bypass is likely due to the high residence time and shallow bathymetry of the region [17,39,40].

The physical habitat attributes of capture locations may also explain the spatial variability in growth along the SFE. Juvenile Delta Smelt caught in wider and deeper channels (Sacramento River and Sacramento Deep Water Ship Channel) exhibited slower growth than fish found in Suisun Bay and small tidal sloughs (Cache Slough, Yolo Bypass). Cache Slough, Yolo Bypass, and Suisun Bay are fringed with tidal marsh habitats while the Sacramento Deep Water Ship Channel and Sacramento River are highly modified, dredged channels with shorelines reinforced with rip-rap. Zooplankton abundance in areas disconnected from tidal marsh would likely be driven by in-situ production, while habitats connected to tidal marsh may receive additional zooplankton inputs from marsh-derived productivity during ebb tides [69].

The Yolo Bypass adds to the environmental heterogeneity within the SFE and thus may promote greater demographic life history diversity. For example, due to the shallow backwater nature of the Yolo Bypass, water temperature warmed earlier in this region. This may provide the Delta Smelt population as a whole a broader spawning period, as hatch dates were consistently earlier in the Yolo Bypass relative to other locations in the SFE. Female Delta Smelt have been documented to produce multiple clutches of eggs within a season with a relatively short refractory period, and number of egg clutches is a key factor in the total egg production for the species [70]. Thus, habitats that prolong the period of time when temperatures are optimal for spawning and hatching may facilitate higher production of cohorts within a season, providing population resilience.

Despite the potential benefits that the Yolo Bypass may offer Delta Smelt, uncertainties remain regarding its future use by the species. For example, a number of invasive species have increased in abundance within the San Francisco Estuary [71,72]. Mississippi Silverside (*Menidia audens*), a small introduced fish species and predator of larval Delta Smelt [73], has been increasing in number throughout the littoral habitat of the Sacramento-San Joaquin Delta [74] and is now one of the most common fish species encountered in the Yolo Bypass [39,40]. Wakasagi (*Hypomesus nipponensis)*, a congener to Delta Smelt introduced from Japan, has also been observed more frequently in the Yolo Bypass in the past few years [50]. Though hybridization between the two species is rare and appears to be unidirectional towards Wakasagi [48,50], it may lead to wasted reproductive efforts for Delta Smelt. Lastly, while juvenile Delta Smelt can tolerate up to 27–28°C in a laboratory setting [75] and up to 25°C in the field [43], adult Delta Smelt seem to require a lower optimal temperature for long-term survival and spawning [75,76]. Future warming of the Yolo Bypass due to climate change may shorten the maturation and spawning windows of Delta Smelt in the spring and exclude the species from the region entirely in from late-spring to fall [16,56].

Unlike other native fish species in the San Francisco Estuary that utilize inundated floodplain habitat [10,13], Delta Smelt does not appear to use the Yolo Bypass when flood events occur. Instead, Delta Smelt seem to occupy habitat further downstream in the Estuary during periods of high flow [46] and showed greatest use of the Yolo Bypass during dry periods when it exists primarily as a tidal slough. The Yolo Bypass acts as a unique habitat for Delta Smelt that produces distinct hatch date cohorts and provides high growth nursery habitat, which collectively may promote population resilience for this endangered species. However, our results also indicate that no single region or habitat provided the best rearing opportunity for juvenile Delta Smelt across different years with different climatic conditions. A complex mosaic of habitats that incorporates floodplain-tidal slough environment such as the Yolo Bypass will likely be needed to promote the life history diversity and resiliency of this declining estuarine fish species.

## Supporting information

S1 Fig Map of the upper San Francisco Estuary showing the locations where Delta Smelt were sampled and caught in between June and August for each year from 2010 to 2016 by the California Department of Fish and Wildlife’s Summer Townet Survey (Yolo Bypass rotary screw trap catch not shown). (DOCX)

S2 Fig Estimated volume of freshwater inflow into the Sacramento-San Joaquin Delta in cubic meter per second for all water years between 2010 and 2016 (for methods, see: https://www.water.ca.gov/Programs/Environmental-Services/Compliance-Monitoring-And-Assessment/Dayflow-Data). Water year in California begins in October and ends in September. For example, water year 2010 begins in October 2009 and ends in September 2010.
(DOCX)

S1 Dataset Juvenile Delta Smelt size, growth, and age data from 2010 to 2016 for five regions within the San Francisco Estuary. Region codes are as follows: Cache = Cache Slough region, DWSC = Sacramento Deep Water Ship Channel, LSZ = low salinity zone, SR = Sacramento River, Yolo = Yolo Bypass.
(CSV)

S2 Dataset Diet data from juvenile Delta Smelt collected at the Yolo Bypass Toe Drain from 2010 to 2015. Weight is measured in grams.
(CSV)
