# Supplementary material for: Role of freshwater floodplain-tidal slough complex in the persistence of the endangered delta smelt
Source: PLoS One. 2019 Jan 2;14(1):e0208084. doi: 10.1371/journal.pone.0208084 (PMC6314582; doi:10.1371/journal.pone.0208084)

**S1 Fig.** Maps of the upper San Francisco Estuary showing the locations where Delta Smelt were sampled and caught in between June and August for each year from 2010 to 2016 by the California Department of Fish and Wildlife’s Summer Townet Survey (Yolo Bypass rotary screw trap catch not shown).
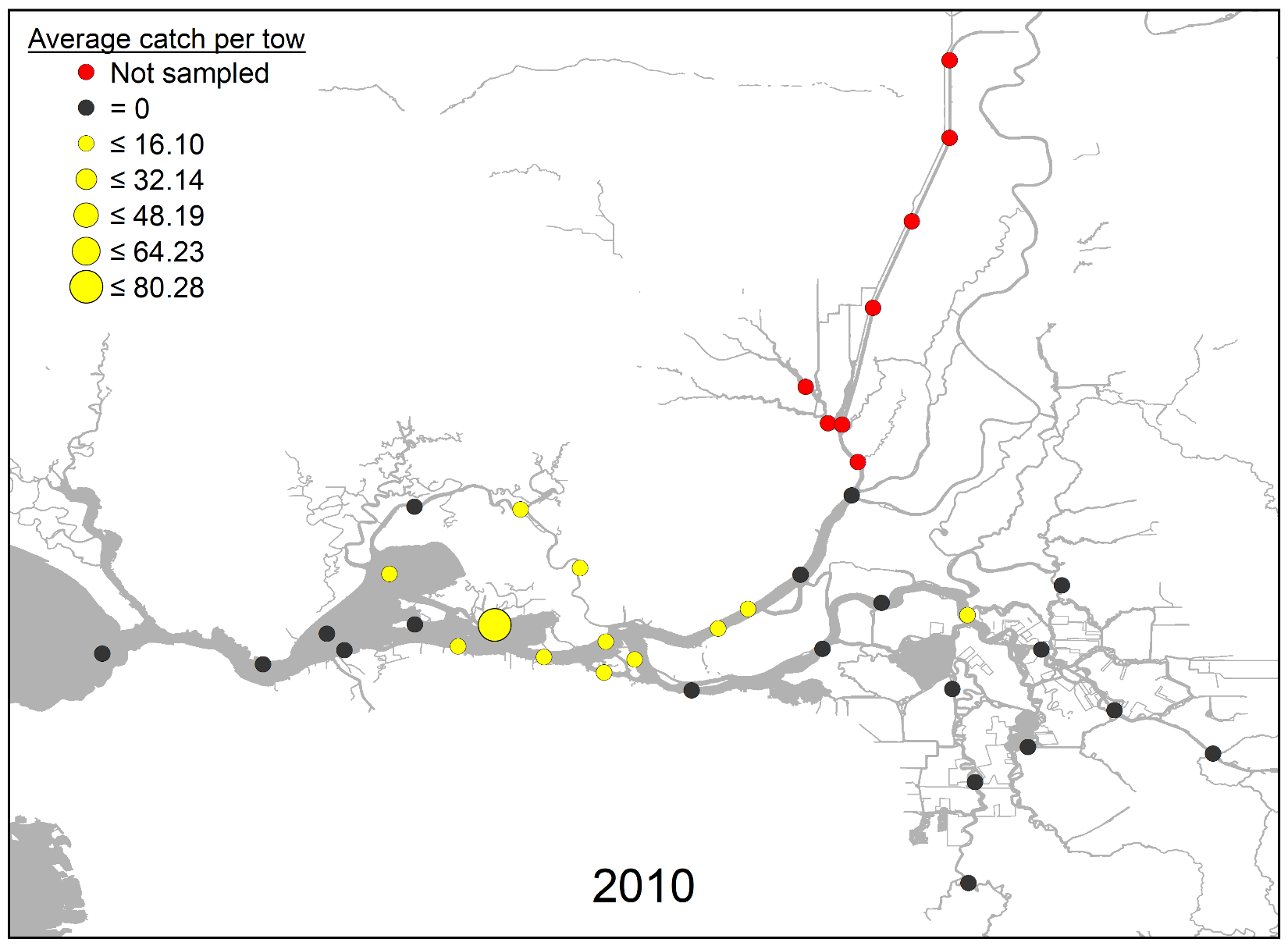

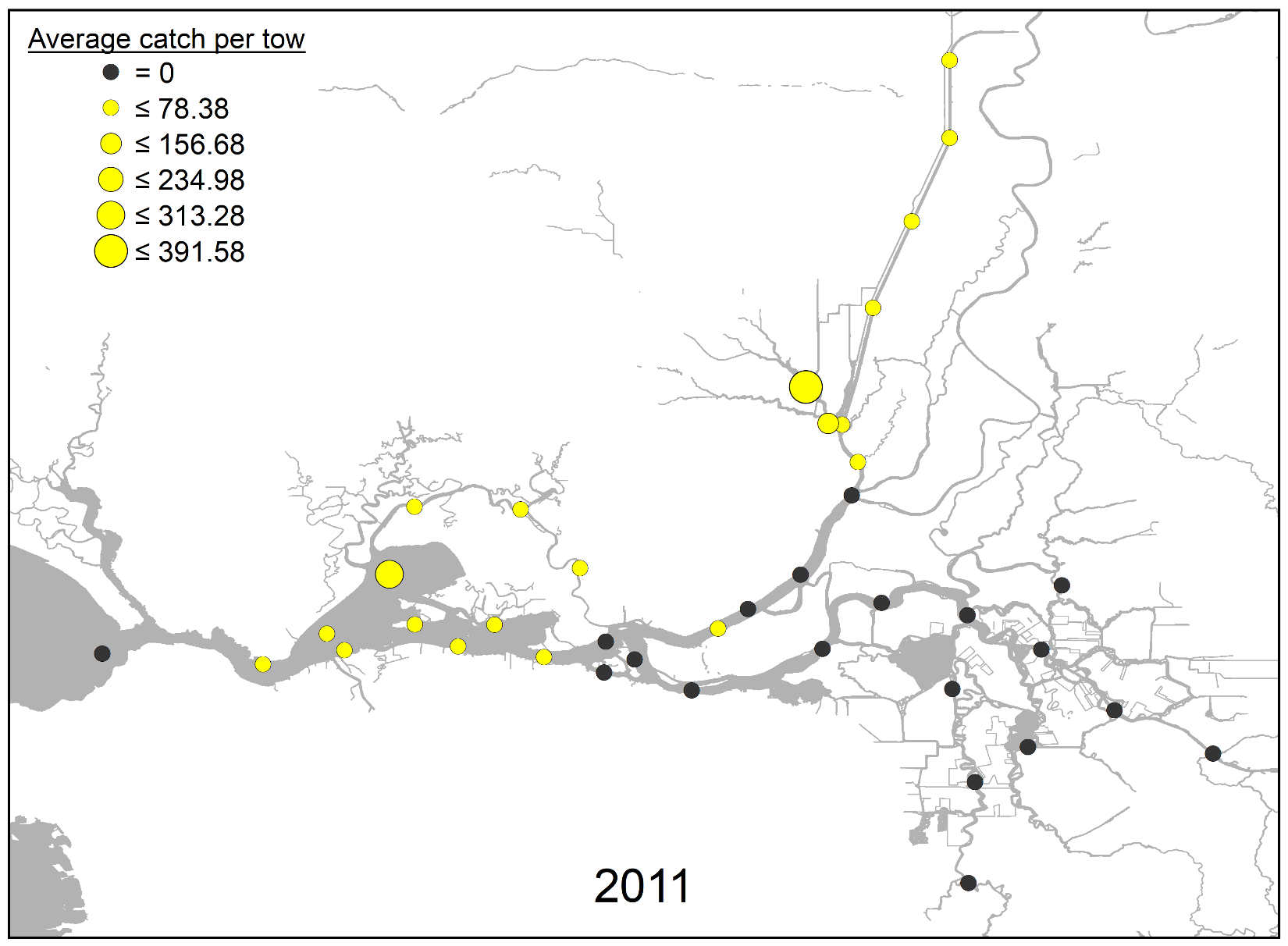

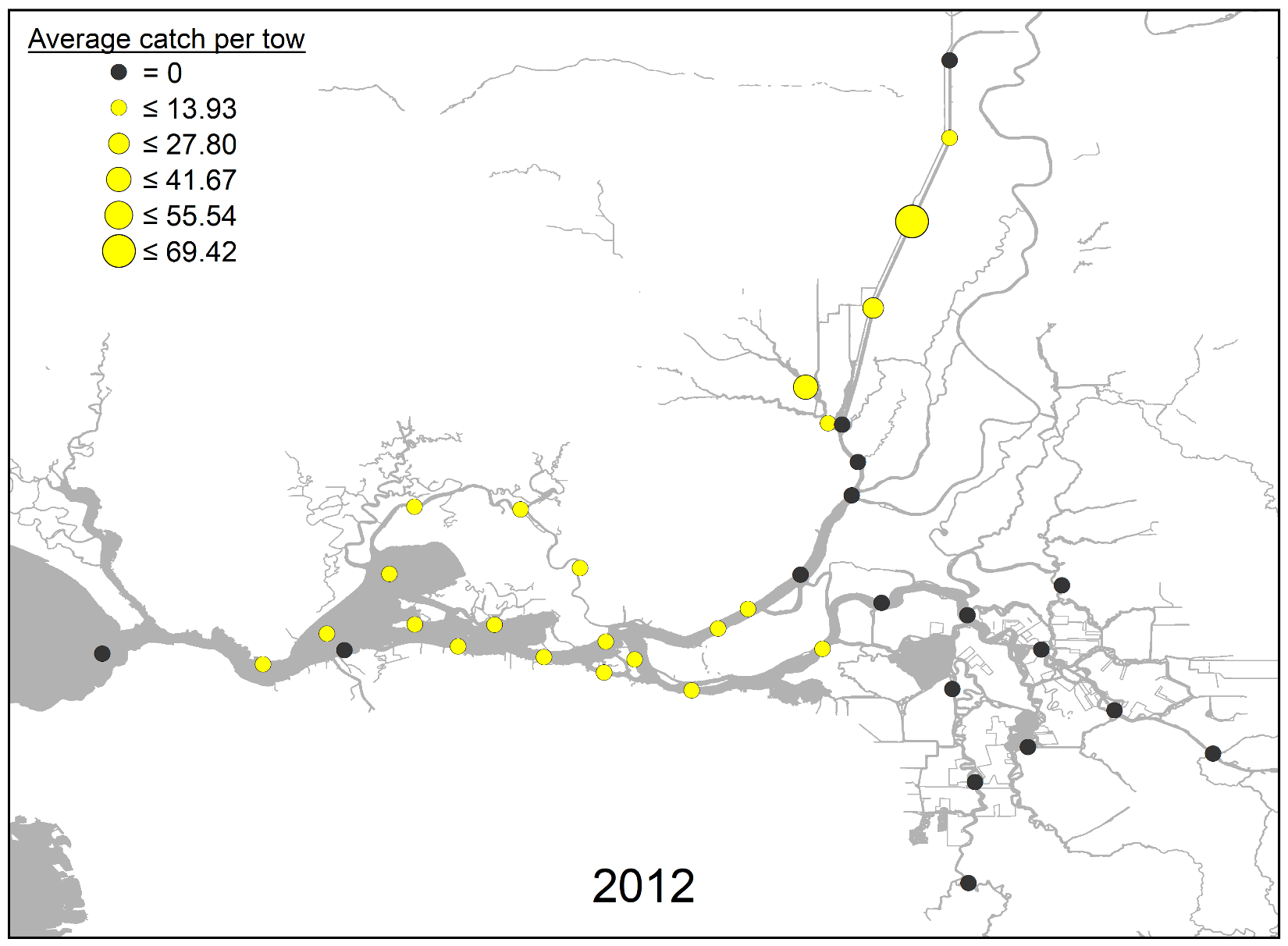

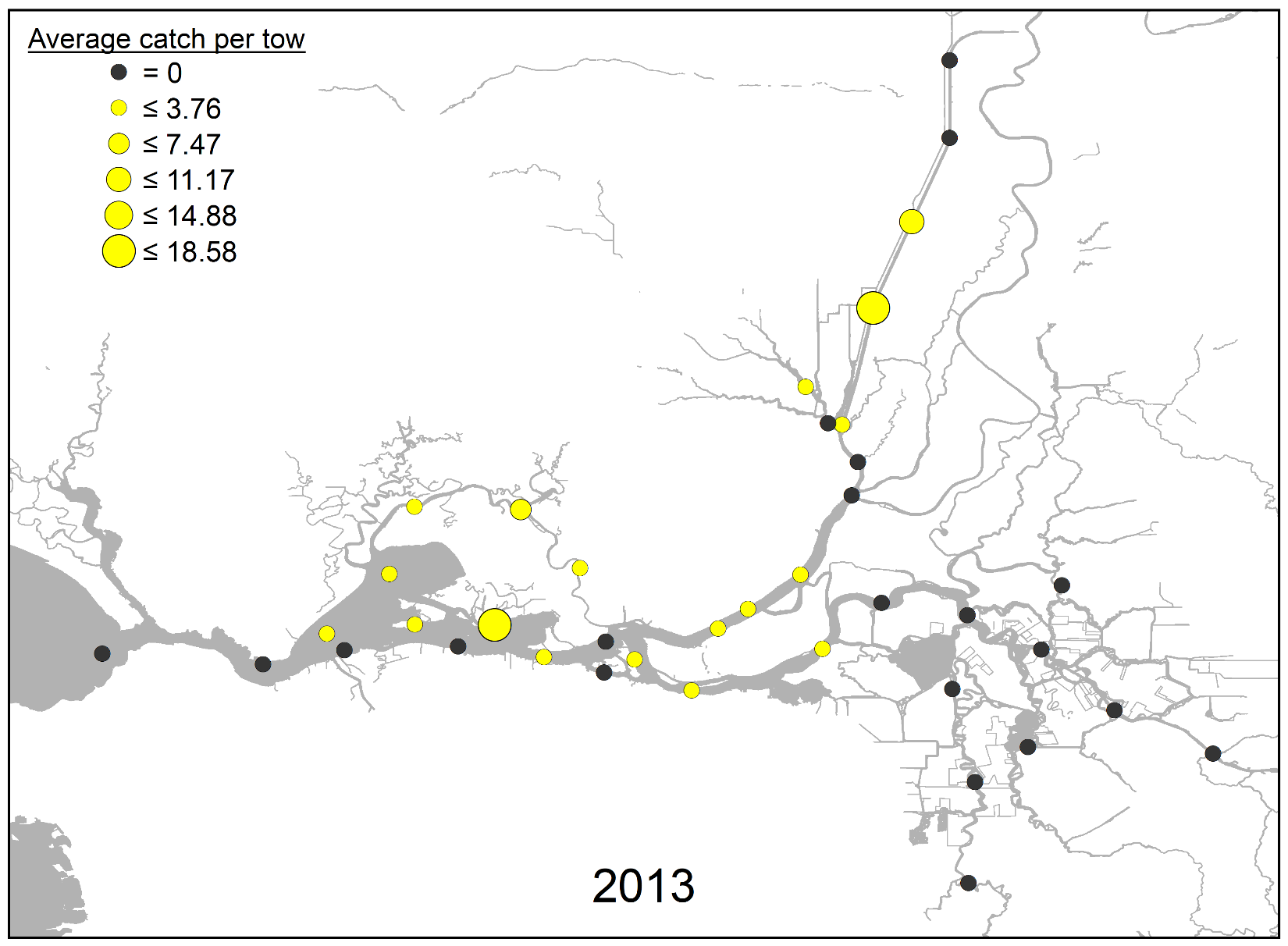

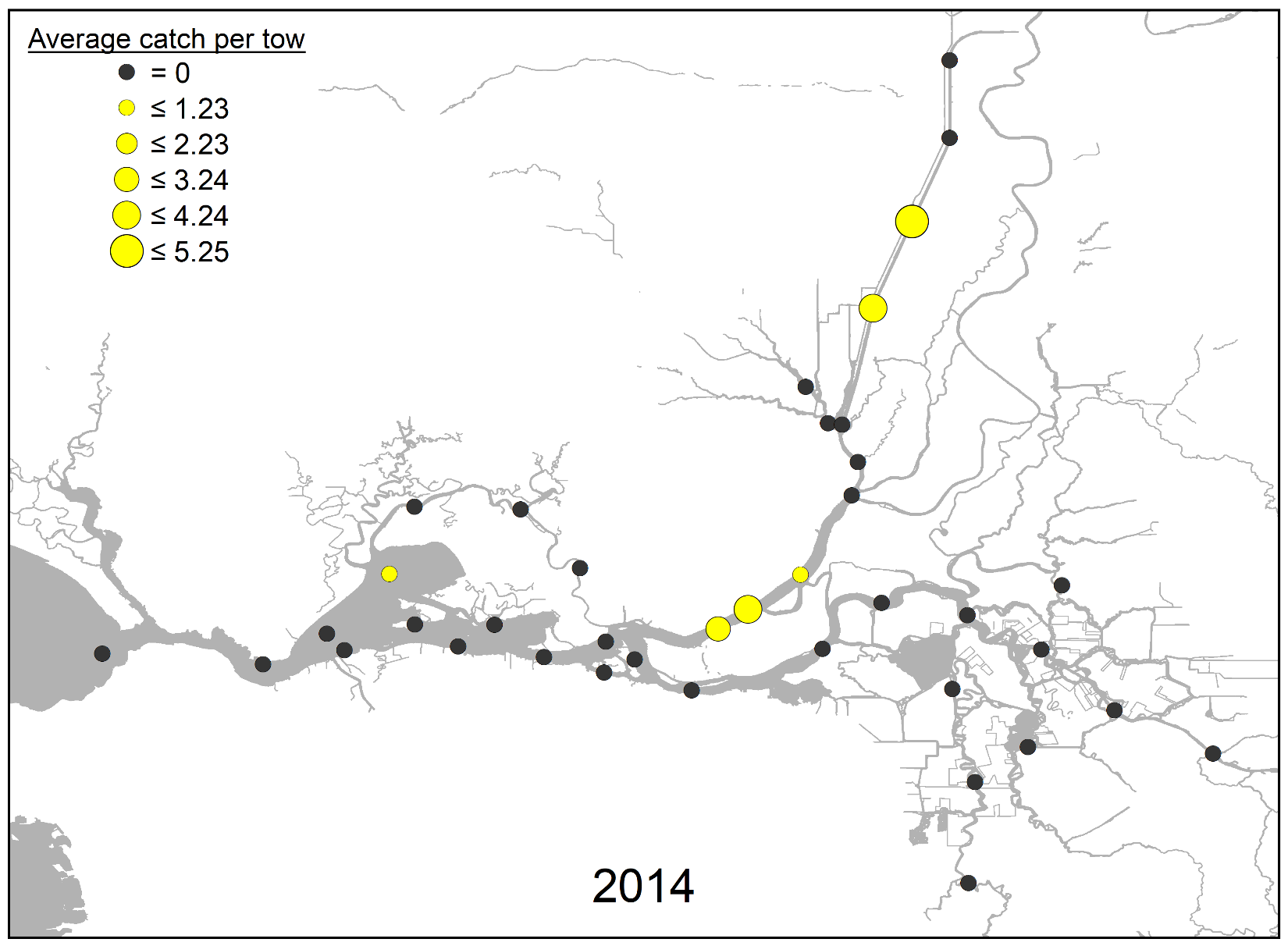

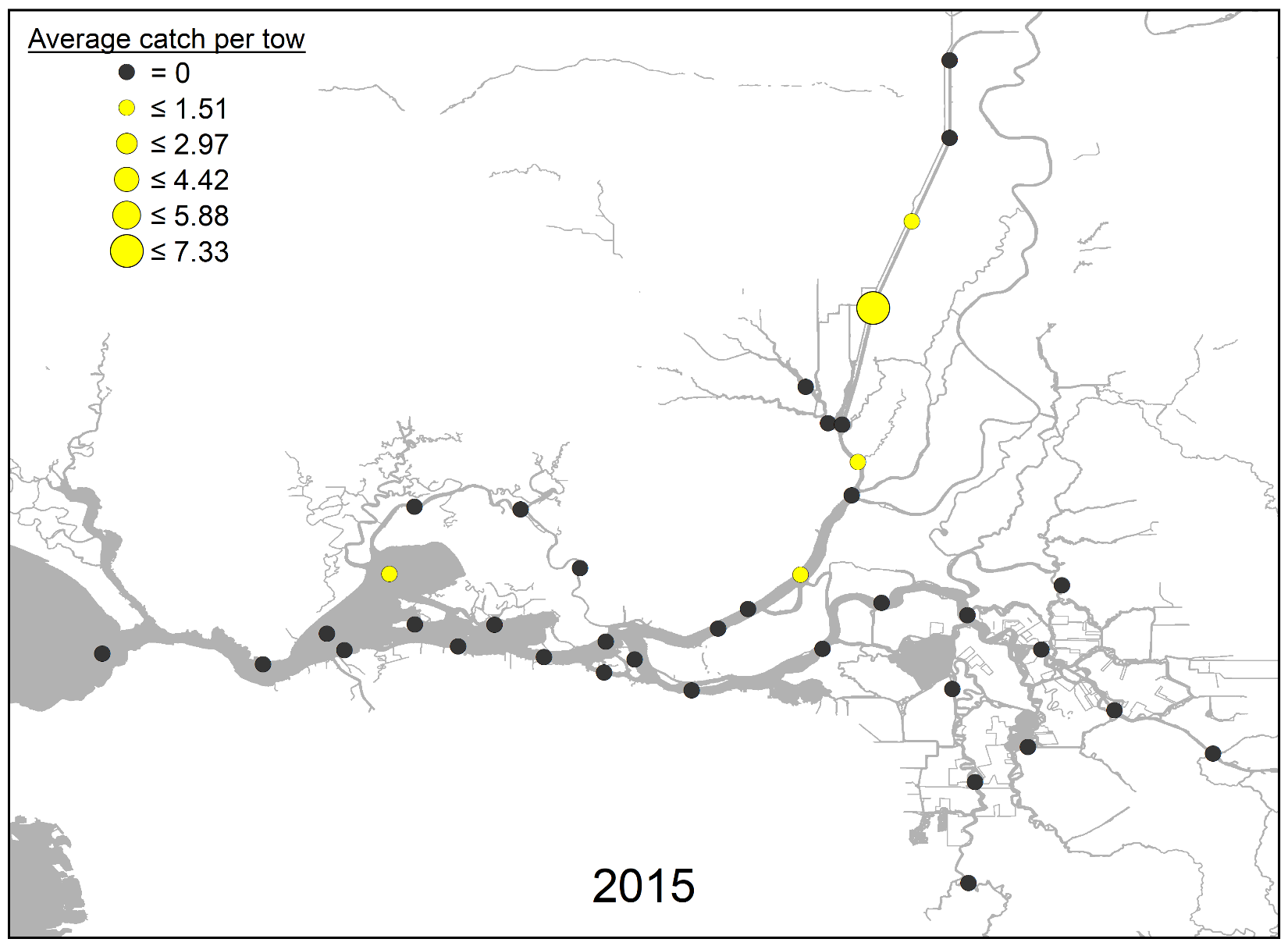

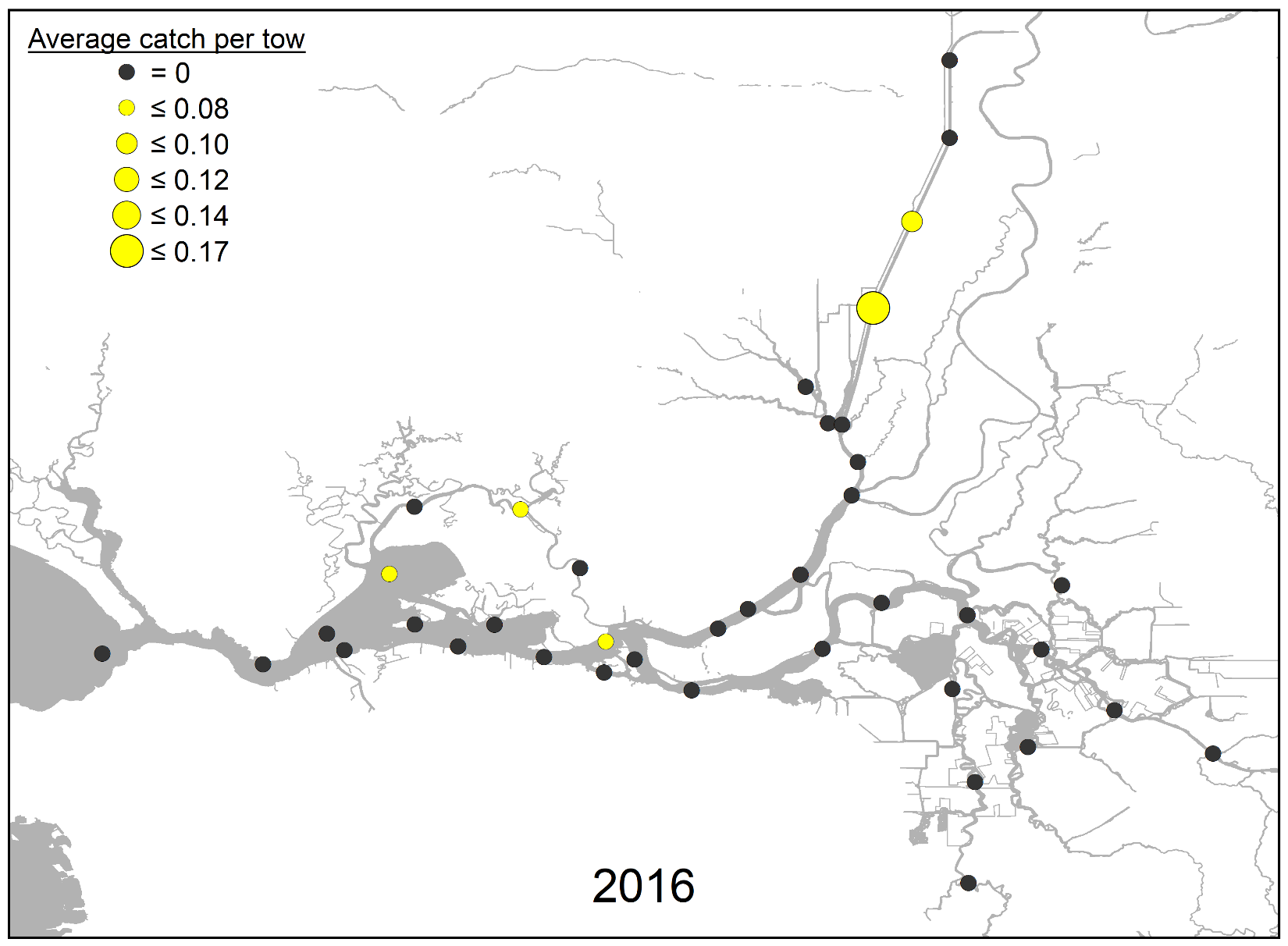

Supplement: S1 Fig — (DOCX) [file pone.0208084.s001.docx]
