# Supplementary material for: Role of freshwater floodplain-tidal slough complex in the persistence of the endangered delta smelt
Source: PLoS One. 2019 Jan 2;14(1):e0208084. doi: 10.1371/journal.pone.0208084 (PMC6314582; doi:10.1371/journal.pone.0208084)
Supplement: S2 Fig — Water year in California begins in October and ends in September. For example, water year 2010 begins in October 2009 and ends in September 2010. (DOCX) [file pone.0208084.s002.docx]

**S2 Fig.** Estimated volume of freshwater inflow into the Sacramento-San Joaquin Delta in cubic meter per second for all water years between 2010 and 2016 (for methods, see: <https://www.water.ca.gov/Programs/Environmental-Services/Compliance-Monitoring-And-Assessment/Dayflow-Data>). Water year in California begins in October and ends in September. For example, water year 2010 begins in October 2009 and ends in September 2010.**
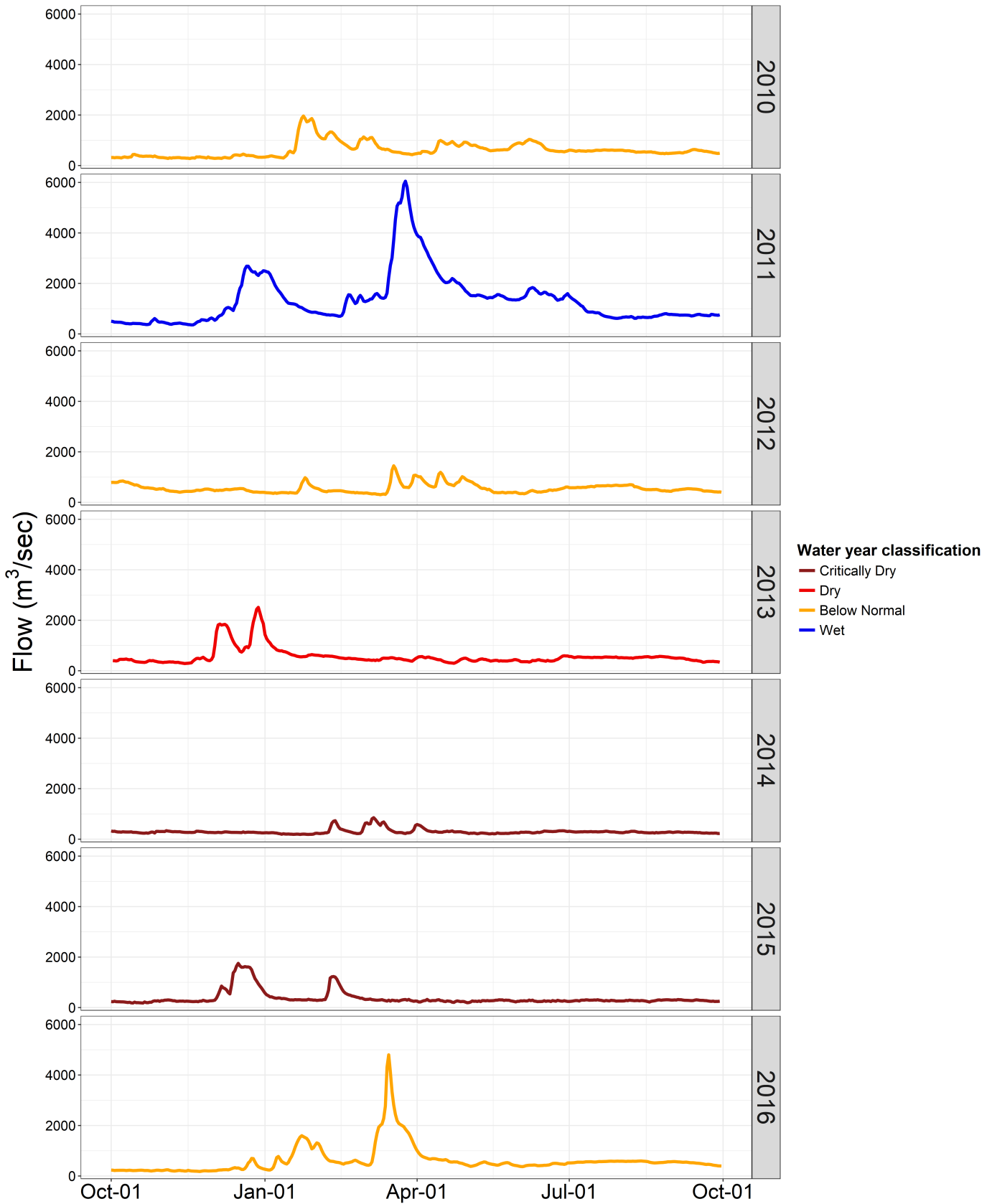
**
